# Complexity of Interferon-γ Interactions with HSV-1

**DOI:** 10.3389/fimmu.2014.00015

**Published:** 2014-02-06

**Authors:** Nancy J. Bigley

**Affiliations:** ^1^Microbiology and Immunology Program, Department of Neuroscience, Cell Biology and Physiology, Wright State University, Dayton, OH, USA

**Keywords:** HSV-1, suppressors of cytokine signaling 1 and 3, Tregs, trigeminal ganglion, interferon-gamma

## Abstract

The intricacies involving the role of interferon-gamma (IFN-γ) in herpesvirus infection and persistence are complex. Herpes simplex virus type 1 (HSV-1) uses a variety of receptors to enter cells and is transported to and from the host cell nucleus over the microtubule railroad via retrograde and anterograde transport. IFN-γ exerts dual but conflicting effects on microtubule organization. IFN-γ stimulates production of suppressors of cytokine signaling 1 and 3 (SOCS1 and SOCS3), which are involved in microtubule stability and are negative regulators of IFN-γ signaling when overexpressed. IFN-γ also interferes with the correct assembly of microtubules causing them to undergo severe bundling, contributing to its anti-viral effect. Factors leading to the decision for a replicative virus lytic cycle or latency in the trigeminal ganglion (TG) occur on histone 3 (H3), involve IFN-γ produced by natural killer cells and non-cytolytic CD8^+^T cells, SOCS1, SOCS3, and M2 anti-inflammatory microglia/macrophages maintained by inhibitory interleukin 10 (IL-10). Both M2 microglia and CD4^+^CD25^+^Foxp3^+^ Treg cells produce IL-10. Histone deacetylases (HDACs) are epigenetic regulators maintaining chromatin in an inactive state necessary for transcription of IFN-γ-activated genes and their anti-viral effect. Following inhibition of HDACs by stressors such as ultraviolet light, SOCS1 and SOCS3 are acetylated, and chromatin is relaxed and available for virus replication. SOCS1 prevents expression of MHC class 1 molecules on neuronal cells and SOCS3 attenuates cytokine-induced inflammation in the area. A model is presented to unify the effects of IFN-γ, SOCS1, SOCS3, and HSV-1 on H3 and chromatin structure in virus latency or reactivation. HSV-1 latency in the TG is viewed as an active ongoing process involving maintenance of microglia in an M2 anti-inflammatory state by IL-10. IL-10 is produced in an autocrine manner by the M2 microglia/macrophages and by virus-specific CD4^+^Foxp3^+^ Treg cells interacting with virus-specific non-cytolytic CD8^+^ T cells.

## Introduction

Progression and recrudescence of herpes simplex virus type 1 (HSV-1) infection are intimately involved with IFN-γ. The interactions of HSV-1 and IFN-γ with the host cell cytoskeletal network and the nuclear epigenetic changes involving histone-3 (H3) are examined in lytic and latent infection.

IFN-γ has been studied primarily as an immunomodulatory molecule in macrophages, dendritic cells, and lymphoid cells (1, 2). The majority of investigations concerning the effects of IFN-γ on the pathogenesis of HSV-1 involve macrophages and other immune cells (3, 4). Although the effects of IFN-γ on non-lymphoid cells are not well established, many non-lymphoid cells in human tissues express receptors for IFN-γ (5). The IFN-γ receptor (IFNGR) is distinctly expressed by endothelial cells and certain epithelial cells. This review focuses on the effects of IFN-γ on the cellular events in the pathogenesis of HSV-1 from initial infection in epithelial cells, especially keratinocytes, to latent infection in trigeminal neurons.

Since initial infection of humans with HSV-1 is usually unnoticed, extrapolation of observations occurring in murine models and tissue cultures will be used to portray these events. This review focuses on:

1. Cellular receptors for IFN-γ and for HSV-1 and the cytoskeletal effects of receptor ligation.
2. Epithelial and neuronal cells involved in innate resistance to HSV-1 and the cytoskeletal effects including intracellular involvement of pattern recognition receptors (PRRs).
3. Host cell resistance in latency and recurrent infection.
  - Receptor ligation.
  - Modulating cytokines in latency and recurrent infection.

## Cellular Receptors for IFN-γ and HSV-1

A heterodimer consisting of two chains, IFNR1 and IFNR2, constitutes the IFNGR. Binding of IFN-γ to IFNGR1 induces the rapid dimerization of each IFNGR1 chain, forming a recognition site for the extracellular domain of each IFNGR2. The intracellular regions of this IFN-γ-IFNGR complex bring together inactive JAK1 and JAK2 kinases, which transactivate each other and phosphorylate IFNGR1, forming a paired set of STAT1 docking sites on the ligated receptor. After binding in close proximity with JAK kinases, the STAT1 molecules are phosphorylated at tyrosine 701, which activates the STAT molecules to dissociate from the receptor complex form homodimers and translocate to the nucleus as specific gene activators (6). Alternately, Johnson et al. (7) obtained evidence that suggests a different scenario in which the IFNGR1 chain is complexed to activated STAT1 homodimer and activates JAKs to bind to a specific sequence in the promoter region of immediate early (IE) IFN-γ-inducible genes effecting transcription. The activated JAKs are involved in specific epigenetic events such as phosphorylation of tyrosine 41 on histone H3. In turn, this results in dissociation of histone inhibitor protein α1 from histone H3, exposing euchromatin for specific gene activation (7). The Johnson model is more satisfying intellectually in explaining the specificity of the transcription factor for the target gene; protein sequences in the IFNGR1 chain would lead the complex to bind to complementary sequences in a protein associated with the specific target gene.

Herpes simplex virus type 1 initially infects epithelial cells, specifically keratinocytes. Dynamin, a microtubule GTPase mediates herpes virus entry into keratinocytes (8). Entry involves both endocytosis and direct fusion at the plasma membrane, processes mediated by dynamin and dependent on cholesterol (8, 9). The various receptors that are known to be involved in HSV-1 entry are listed in Table 1. Virus entry appears to be cell specific. Certain cell lines will permit HSV-1 entry through the low pH endocytic pathway while others exhibit entry through the direct fusion with plasma membrane of the host cell (10).

**Table 1:** **HSV-1 glycoproteins involved in virus attachment and entry (10)**.

HSV-1 glycoprotein	Function	Components
**ATTACHMENT PROTEINS**		
gB and/or gC	Initial attachment	Heparan sulfate proteoglycans (HSPG); abundantly expressed on the surface of almost all cell types
**HSV-1 ENTRY PROTEINS**		
gD	Fusion trigger	HVEM (HveA)
		Nectin-1/nectin-2
		3-*O*-sulfated heparan sulfate proteoglycan (3-OS HS)
gB	Fusogen	Paired immunoglobulin-like type 2 receptor-a (PILRa)
		Myelin-associated glycoprotein (MAG)
		Non-muscle myosin heavy chain IIA (NMHC-IIA)
gH-gL	Fusion regulator	ανβ3 integrin

*HSV-1 and host cell cytoskeletal reorganization mediated by HSV-1 entry, microtubule transport to nuclear pore, and replication of virus*.

## Retrograde Cellular Transport of HSV-1

**Figure 1:**
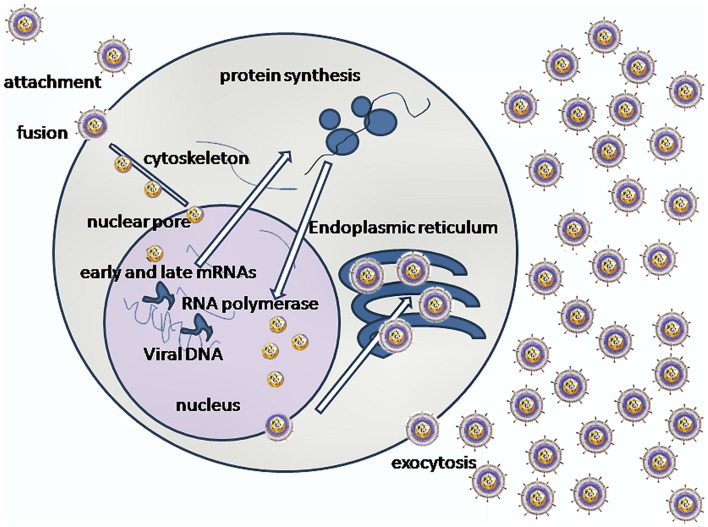
**A simplified version of the complexity of interactions involved in HSV-1 replication is shown (image credit: Graham Colm)**.

Following attachment of the virus by fusion, viral capsids are transported along microtubules to the nuclear pore where the capsid is uncoated and viral DNA is injected into the nucleus (11) (Figure 1). Cytoskeletal rearrangements occur within the infected cell upon binding HSV-1 glycoproteins (12). HSV-1 capsids bind to and traffic along microtubules associated with a dynein–dynactin complex (13). Dynein, a minus end-directed microtubule-dependent motor, binds to the incoming capsids and propels them along microtubules from the cell periphery to the nucleus (14). The VP26 capsid protein appears to be the main candidate for viral binding to the dynein motor of microtubules for retrograde transport to cell nucleus (15). Several tegument proteins (VP1/2 and UL37) remain associated with the capsid, which binds to the nuclear pore complex (NPC). After DNA entry into the nucleus, the capsid with remaining tegument proteins is retained on the cytoplasmic side of the nuclear membrane (16). Virus replication occurs in nucleus (16). Sequential gene expression occurs during replication of HSV-1; the α, IE genes are involved in organizing the transcriptional elements. The β or early phase genes carry out the replication of the viral genome and the βγ/γ late phase genes are involved in expression of structural proteins in high abundance (17). Although the IE α gene regulatory protein ICP27 enhances viral gene expression and is predominately nuclear, it shuttles to the cytoplasm during HSV infection, employing an N-terminal nuclear export signal (NES) (18). ICP27 activates expression of β and γ genes by different mechanisms, it shuts off host protein synthesis; it shuttles between the nucleus and cytoplasm in regulating late protein synthesis (19). HSV-1 major capsid proteinVP5 gene (UL19) is expressed with βγ gene kinetics (20). VP19C is a structural protein of HSV-1 and is essential for assembly of the capsid. It also contains a NES, which permits it to shuttle from the cytoplasm to nucleus for virus assembly (21).

## Anterograde Cellular Transport of HSV-1

Non-enveloped capsids recruit kinesin-1 (a positive end microtubule motor) and dynein to undergo transport to their site of envelopment (13). The ability to move bidirectionally appears to depend on cell type and ensures that the capsids come in contact with the appropriate compartment for further development (13). Microtubule-mediated anterograde transport of HSV-1 from the cell nucleus is crucial for the spread and transmission of the virus (22). The majority of HSV-containing structures attached to the microtubules contain the *trans*-Golgi network marker TGN4 (23). This suggests that HSV modifies TGN exocytosis or sorting machinery, which would accelerate the movement of HSV capsids to the cell surface. Their conjecture is supported by the observation that accumulation of HSV particles in cytoplasm is short-lived. In epithelial cells, 10% of enveloped particles are found in the cytoplasm whereas the remaining 90% of these mature particles are on the cell surface (23).

In live imaging of infected rat or chicken dorsal root ganglia, approximately 70% of live viruses undergo axonal transport (24). The enveloped HSV-1 virions were identified in close association with neural secretory markers and trafficked to amyloid precursor protein (APP)-positive vesicles during anterograde egress. To ensure the proper distribution of the cargo (HSV-1 in this case), both positive and negative motors are attached. APP levels were found to be well-correlated with the amount of the components of each motor on the vesicles (25).

## Significance of Exosomes (Microvesicle/L-Particles) in HSV-1 Infection

Electron cryo-tomography was used to visualize HSV-1 interactions with cultured dissociated hippocampus neurons. These infected cells produced and released both infective virions and non-infectious particles referred to as light (L) particles or exosomes (26, 27). L-particles lack capsids and viral DNA (28–30). Shared assembly and egress pathways were suggested since virions and L-particles formed in close proximity are often associated with clathrin-like coats (26). In contrast to 2D images of 30–100 nm diameter oxosomes (27, 31), HSV-1 infected cultures of human foreskin fibroblasts yielded larger 3D images of L-particles; 280 nm diameter size particles were seen intracellulary and 177 nm diameter particles were found extracellularly (26). The complex virus–host interactions at sites of initial HSV-1 infection permit virus persistence in that these microvesicles may interfere with host protective immune responses, e.g., preventing antibody neutralization of infectious virions.

In summary, the cytoskeletal reorganizations involving initial retrograde transit of HSV-1 to the cell nucleus, where viral replication or latency is initiated, to the anterograde transport and export of replicated virus depend on a myriad of viral and cytoskeletal protein interactions. The exosomes exported during lytic infection add an additional layer of complexity to HSV infections.

## Host Cell Cytoskeletal Reorganization Mediated by IFN-γ

IFN-γ exerts effects on a wide range of cellular programs including: upregulation of an anti-viral state, antigen processing and presentation, microbicidal activity, immunomodulation, leukocyte trafficking and apoptosis, and downregulation of cellular proliferation. It orchestrates many of these cellular effects alone or in conjunction with other cytokines or pathogen-associated molecular patterns (PRRs) or bioactive molecules such as lipopolysaccharide (LPS) from gram-negative bacteria (1, 32). The effects of IFN-γ on the cell’s cytoskeleton are little known. IFN-γ induces a higher basal level of F-actin and activation of Rac-1 (a GPase), which affects cytoskeletal rearrangement resulting in decreased phagocytosis by monocyte-derived macrophages (33). During viral entry, activation of RhoA and Rac-1 results from attachment of Kaposi’s sarcoma-associated herpes virus (KHV or HHV8) glycoprotein B (gB) to integrin α3β1; this leads to acetylation and stabilization of microtubules (12). It is intriguing to speculate that the activation of Rac-1 by IFN-γ may also enhance cytoskeletal reorganization and stabilization of microtubules in HSV-1-infected cells. RhoA and its downstream target Rho kinase are involved in cytoskeletal reorganization in cells infected with other viruses. The Rho family GTPase activity within the host cell triggers microtubule stabilization for viral transport during early infection of African swine fever virus (34).

IFN-γ causes an increase in expression of both class I and class II MHC molecules on the cell surface. Trafficking of MHC class II molecules in antigen-presenting cells is dependent on the cytoskeletal network (35) and is dependent on myosin II, an actin-based motorprotein in B lymphocytes (36). In dendritic cells, the microtubule-based proteins, dynein and kinesin, determine retention and transport of MHC class II-containing compartments to the cell surface (37).

Any further effect of IFN-γ on the cell cytoskeleton involves indirect association with the effects of this molecule on GTPases involved in cell migration (38). IFN-γ inhibits monocyte migration by suppressing actin remodeling of the cytoskeleton and polarization in response to chemokine CCL2, a STA1-dependent process modulating activity of Pyk2, JNK, and the GTPases Rac and Cdc42 (38). Rho kinase (ROCK) is a downstream effector of Rho GTPase and regulates many crucial cellular processes through its control of actin and microtubules (39). In an adenocarcinoma colonic (T84) cell line, IFN-γ treatment activated Rho GTPase that upregulated expression of Rho-associated kinase (ROCK), which then mediated internalization of tight junction proteins from the apical plasma membrane into actin-coated vacuoles; this process was dependent on the ATPase activity of a myosin II motor (40).

Either HSV-1 infection or IFN-γ treatment upregulated expression of suppressor of cytokine signaling 1 (SOCS1) in murine keratinocyte cell lines (41). SOCS1 expression was magnified in IFN-γ-treated HSV-1 infected keratinocytes, reflecting a profound inhibition of the IFN-mediated anti-viral effect in both the cytoplasm and nucleus of infected keratinocytes. Yokota et al. (42) noted that SOCS3 induction varied among cell lines. They observed that HSV-1 rapidly induced expression of SOCS3 in a human amniotic cell line (FLcells) resulting in efficient viral replication. In human monocytic cell lines (U937 or THP1), HSV-1 did not induce SOCS3 expression; a persistent infection producing low virus yields resulted in those cells (42).

**Figure 2:**
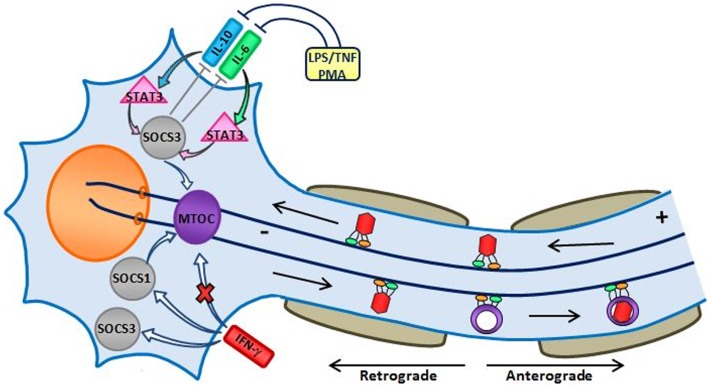
**Hypothetical effect of IFN-γ on microtubules of an HSV-1-infected trigeminal neuron (image credit: Trista D. Smith)**. Herpes simplex virus type 1 invades nerve endings, which is transmitted by microtubule motor proteins via retrograde transport and its DNA is deposited into the nucleus of the cell (47). IFN-γ induces expression of both SOCS1 and SOCS3 (48), but also interferes with the correct assembly of microtubules causing them to undergo bundling (49). Both SOCS1 and SOCS3 promote the stability of the microtubule network (45, 50). In addition, SOCS3 maintains the integrity of the MTOC by anchoring it to the centrosome (45). Cytokines produced by neighboring cells, e.g., IL-6 and IL-10 by macrophages/microglia, stimulate activation of STAT3; STAT3 stimulates a much stronger induction of SOCS3 in response to IL-10 when compared to IL-6 (51).

IFN-γ promotes expression of SOCS1 at the transcriptional level (43). As shown in Figure 2, SOCS1 localizes to the microtubule organizing center (MTOC) (44) as does SOCS3 (45). Both SOCS1 and SOCS3 enhance FAK- and RhoA-activation leading to increased cell adhesion and reduced migration (46).

In summary, IFN-γ exerts anti-viral effects, induces expression and trafficking of MHC class II molecules in antigen-presenting cells, effects actin cytoskeletal reorganization involved in phagocytosis and microtubule destabilized bundle formation. In contrast, IFN-γ contributes to microtubule stabilization by upregulating expression of SOCS1 and SOCS3.

## HSV-1 Lytic Versus Latent Infection

Lytic HSV-1 infection occurs in epithelial cells. As indicated in Table 1, the virus attaches to cell membrane receptors such as heparan sulfate (52), facilitated by viral glycoproteins B (gB) and C (gC) (53). Glycoprotein D (gD) facilitates virus adsorption to the host cell and glycoproteins H and L (gH and gL) are responsible for membrane penetration of the virus into the host cell [reviewed in Ref. (53)]. Furthermore, Dingwell et al. (54) demonstrated that glycoproteins E and I (gE and gI) are responsible for HSV-1 spread from one neuron to another neuron. In lytic infection, virus IE genes (α genes) are expressed first, followed by expression of early β genes, DNA replication, and expression of late γ genes. The maximum rate of synthesis by α genes occurs 3–4 h post infection. The β genes are responsible for the highest rate of synthesis 5–7 h post infection. The synthesis of γ genes increases until 12 h post infection. Use of the protein synthesis inhibitor, cycloheximide, confirmed that IE polypeptides expression occurs without prior viral protein synthesis (55). The IE genes consist of ICP0, ICP4, ICP22, ICP27, ICP47, and Us1.5 (56). Wysocka and Herr (57) revealed that IE genes have VP16-response elements (VRE). In latency, a single transcript is generated, which encodes a precursor for four distinct HSV miRNAs, which act to suppress virus replication (58).

In the establishment phase of latency, the virus enters the neuronal cell in which the viral genome remains transcriptionally quiescent. The integrity of the neuron is not compromised, as the cytopathic effect of the productive infection does not occur (59). During establishment of latent infection, gene expression is limited to a gene located within the long repeat elements of the viral genome. Transcription of this gene results in generation of the latency-associated transcripts (LATs) (60). The LAT transcripts (RNAs) have open reading frames; however, the detection of a protein encoded by the LATs has not been observed (58, 61). LAT expression is not an absolute indication of latency establishment (62), as LAT-defective HSV-1 can establish latent infection in mice (28). In contrast, Thompson and Sawtell (63) found that the LAT gene plays a role in establishment of latency, but LAT has no direct role in the HSV-1 reactivation. They found that approximately 30% of the trigeminal ganglion (TG) neurons in mice infected with LAT^+^ HSV-1 harbored latent virus, but only 10% of the neurons in mice infected with LAT-null viruses were positive for HSV-1 DNA. LAT expression has no demonstrable effect on neuronal cell survival at 3 and 31 days after infection with defective HSV-1 (thymidine kinase-deleted) mutants (64). LAT expression was not necessary for cell survival during TK-deleted virus infection.

Establishment of latency may result from the inability of IE genes to induce lytic infection. Marshall et al. (65) showed that HSV-1 established latency in mice in the presence of impaired IE gene expression and the latency was not affected by restoration of VP16, ICP0, or ICP4 coding sequences. These observations suggest that the latency is increased when IE gene expression is inadequate to initiate the lytic infection. The presence of HSV-1 DNA in the nucleus of infected neurons is an important factor for HSV-1 to establish latency (56). During latency, the role of VP16 to initiate lytic gene expression may be inhibited by a defect in the VP16 transport from nerve endings to the neuronal cell body, or due to the presence of this protein in reduced amounts in the neurons (66). Two competitive inhibitors for transcription of VP16, namely the octamer-binding protein (Oct-2) (67) and N-Oct3 (68) compete with VP16 for binding to an α gene promoter. VP16 fails to form a complex with HCF-1 in the Golgi apparatus of sensory neurons. The HCF-1 protein moves to the nucleus upon reactivation of HSV-1 *in vitro* (69). In humans, HSV-1 reactivation can be spontaneous or results from exposure to ultraviolet (UV) irradiation, emotional stress, fever, or immune suppression. Reactivation causes shedding of the virus transported through neuronal axons to the epithelial cells where it can replicate and start a lytic cycle. Hyperthermia efficiently induced HSV-1 reactivation from latency in a few neurons of the TG in infected mice (70). In latency, a single transcript is generated, which encodes a precursor for four distinct HSV miRNAs, which act to suppress virus replication (71).

## Immune Response to HSV-1

Initial host responses to viral infection include production of interferons-α/β by the first cells infected, IFN-γ by human natural killer (NK) cells recognizing the gB and gC of virus-infected targets (72), and proinflammatory cytokines and chemokines by monocytic cells (73). Viruses are recognized by the innate immune system through PRRs such as the Toll-like receptors (TLRs). HSV virions are recognized by the cell membrane TLR2 and intracellular HSV genomic DNA is recognized by the cytoplasmic TLR9. Dendritic cells recognize HSV using both TLR2 and TLR9 (74). Virus-induced IFN-α and IFN-γ are products of human peripheral mononuclear leukocytes (PML) exposed to UV and light-inactivated HSV (75). In the innate response to HSV-2, TLR2 and TLR9 restrict viral load in the brain by synergizing to induce an early cytokine (type I IFN, IL-6, IL-12, RANTES) and cellular responses (76, 77). In mice lacking both TLR2 and TLR9, HSV induces uncontrolled virus replication and lethal encephalitis (77).

## The Role of Exosomes (Microvesicles or L-Particles) in HSV-1 Immunity

Both B cell and T cell immune responses develop during primary viral infection. However, early viral evasion strategies interfere with complete elimination of virus and permit persistence of HSV-1. During HSV-1 infection, microvesicles/exosomes containing viral tegument proteins and glycoproteins, some of which are early transcription factors, are released. Because these virus-like vesicles lack both the viral capsid and DNA, they cannot produce a replication-infective cycle, but can interfere with immune elimination of virus (29, 30, 78). Also, the viral envelope gB is involved in inhibiting the MHCII molecule antigen-processing pathway by coupling with HLA-DR and shunting the complex through microvesicles/exosomes instead of the cell surface (31). This capture of the gB-HLA-DR complex puts complexes into the cellular microenvironment to induce tolerance in bystander T cells (27, 31).

## Immune Effector Cells and Latency

An understanding of the mechanisms that control the HSV-1 latency is elusive. Reactivation from latency is associated with pathological disease due to shedding of the reactivated virus from the sensory ganglia (79). CD8^+^ T cells can inactivate HSV-1 without inducing neuronal apoptosis. It was shown that CD8^+^ T cell lytic granules, granzyme B, can destroy the HSV-1 IE protein, ICP4, which acts as transactivator of β genes required for viral DNA replication. HSV-1 latency is accompanied by chronic inflammation without neuronal damage (80). Trigeminal ganglia latently infected with HSV-1 are infiltrated with CD3^+^ and CD8^+^ T cells, CD68-positive macrophages, IFN-γ, tumor necrosis factor-α (TNF-α), IP-10, and RANTES. These observations suggest that the presence of the immune cells and elevated levels of cytokines within the latently infected trigeminal ganglia are responsive to the clinical use of immunosuppression drugs and subsequent reactivation of virus in the cranial nerves. Immune cell infiltration in latently infected trigeminal ganglia may occur in response to spontaneous reactivation of some neurons leading to expression of HSV-1 lytic cycle transcripts (81). Because of the absence of detectable virus in latently infected TG, this process was referred to as spontaneous molecular reactivation.

CD8^+^ T cells and macrophages/microglia and their cytokine, TNF-α, exert a role in maintaining HSV-1 latency in the trigeminal ganglia. However, NK cells and γδ T cells and their production of IFN-γ play a role in preventing viral replication during the lytic infection (82). HSV-specific CD8^+^ T cells migrate to and are retained in the ophthalmic branch of the TG after intraocular infection (83). In the absence of replicating virus, HSV-1-specific CD8^+^ T cells remain active, secreting IFN-γ in the latent TG. The activated virus-specific memory CD8^+^ T cells, expressed the CD94-NK cell receptor subfamily G2a inhibitory molecule. These cells were not cytotoxic for the Qa-1^b^-expressing neuronal targets, of which there were many in the HSV-1 latent TG. When the Qa-1^b^/CD94-NKG2a interaction was blocked in *ex vivo* experiments, neuronal lysis occurred. Since TGF-β1 can induce expression of the inhibitory CD94-NKG2a molecules, the source of bioactive TGF-β1in the latent TG was attributed to CD4^+^Foxp3 Treg cells also present in the latent TG (83). These observations indicate the presence of a regulatory system that protects irreplaceable neurons from immune destruction (83). Qa1 expression, whether on neurons or lymphoid cells present in the TG, is protected; binding of CD94/NKG2a to Qa1 on activated CD4^+^ T cells provides protection from NK cell-mediated lysis (84).

## IFN-γ and HSV-1 Induce Expression of SOCS1

SOCS1 expression in response to IFN-γ by sensory neuronal cells, but not by microglia, is responsible for the lack of expression of class I MHC molecules by sensory neurons (85). HSV-1 can evade the immune response by SOCS1 expression (41). HSV-1 is resistant to anti-viral effect of IFN-γ in keratinocytes, the major cell replicating virus in recurrent lytic infection. HSV-1-infected keratinocytes exhibit high levels of SOCS1 mRNA and protein expression by preventing STAT1α activation in response to IFN-γ signaling. In this same study, viral ICP0 was involved in activating host cell SOCS1 gene; i.e., both IFN-γ and HSV-1 induced expression of SOCS1 in keratinocytes (41).

The conundrum involving the association and interactions of histones, HSV-1, IFN-γ, and SOCS1/3 in herpesvirus infection and latency is intriguing. Protein acetylation is important in herpesvirus infection as well as in activation of IFN-γ-stimulated genes. Histone acetylation determines how tightly the DNA is wound around the histones. In histones H3 and H4, chromatin is relaxed and accessible to the transcriptional proteins and subsequent increase in gene transcription. In areas of hypoacetylation, chromatin is condensed and genes are silenced (86). Histone deacetylases (HDACs) are transcriptional and epigenetic regulators controlling HSV-1 infection (87). Trichostatin A (TSA), an HDAC inhibitor, suppresses JAK2/STAT3 or JAK3/STAT3 signaling by inducing the promoter-associated histone acetylation of SOCS1 and SOCS3 (88). TSA treatment causes a relaxation of the chromatin structure, a process essential for initiation of transcription. Induction of SOCS1 and SOCS3 expression by TSA is associated with an increase in acetylation of H3 and H4 histone proteins in colorectal cancer cells (88). TSA treatment of HSV-1-quiescently infected neurons induces a productive lytic infection (89). HDAC is essential for the transcriptional activation of IFN-γ-stimulated genes and for host anti-viral immune responses; TSA treatment of cell cultures (HepG2, Huh7, and HeLa cells) promoted the proteasomal degradation of IFN regulatory factor 1 (IRF-1) (90). These observations contribute to a deeper understanding of the fact that IFN-γ is essential in the TG to maintain virus latency. When IFN-γ is neutralized by specific antibody, virus replication occurs in the brain stem of latently infected mice (3).

**Figure 3:**
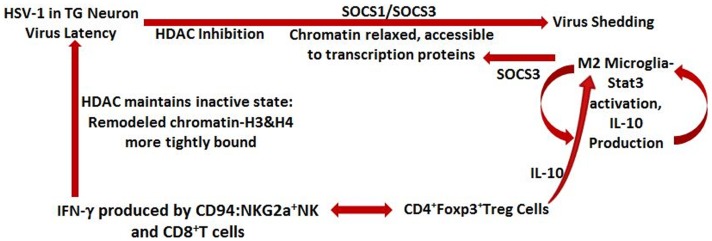
**Schematic representation of events contributing to HSV-1 latency and lytic cycle**. IFN-γ secreted by CD94: NKG2a NK cells and virus-specific, but non-lytic, CD8^+^T cells maintain virus in the latent state; HDAC maintains chromatin in an inactive state and is necessary for transcription of IFN-γ-activated genes and for its anti-viral effect. When HDAC is inhibited by stressor (e.g., UV light), SOCS1 and SOCS3 are acetylated, chromatin is relaxed and accessible for virus transcription, and virus is shed. SOCS1 prevents expression of MHC class 1 molecules on neuronal cells and SOCS3 is involved in attenuating cytokine-induced inflammation in the area. Stimulated M2 microglia produce SOCS3 as well as the immunosuppressive molecule – IL-10, which is also produced by virus-specific CD4^+^ Foxp3^+^ Treg cells. IL-10 exerts a local protective anti-inflammatory effect by maintaining the microglia/macrophages in the M2 anti-inflammatory state in which SOCS3 expression predominates.

When SOCS1 level is elevated over that of SOCS3 in macrophages/microglia, an inflammatory M1 phenotype exists secreting inflammatory cytokines (Bigley et al., unpublished observations) and when SOCS3 predominates in these cells, an anti-inflammatory phenotype exists (91). SOCS3 is involved in attenuating the cytokine-induced inflammatory response in macrophages and microglia by production of endogenous IL-10 and STAT3 activation (92). These observations are illustrated in Figure 3.

## Conclusion

The actin–microtubule cytoskeletal reorganizations that occur in response to HSV-1 infection permit retrograde and anterograde transit of HSV-1 in lytic infection as well as the epigenetic changes that occur in HSV latent and lytic infections. IFN-γ suppression of actin remodeling of the cytoskeleton may influence its anti-viral effect. Cytoskeletal reorganizations involved in retrograde transport of HSV-1 to the neuronal cell nucleus, where viral replication or latency is initiated, to the anterograde transport and export of replicated virus depend on a variety of viral and cytoskeletal protein interactions. A unifying model is proposed to explain latency and emergence from latency at histone H3 sites in nuclei of sympathetic neurons as an active ongoing process. Maintenance of latency involves intimate interactions among immune cells, virus-specific non-lytic CD8^+^ cytotoxic T cells and CD4^+^CD25^+^Foxp3^+^ Treg cells, and M2 microglia. HSV-1 latency occurs when HDAC maintains chromatin in an inactive state permitting IFN-γ produced by NK cells and non-cytolytic CD8^+^ T cells to exert its anti-viral effect. The anti-inflammatory state of the M2 microglia/macrophages is maintained by IL-10 produced by the SOCS3-producing M2 microglia/macrophages and by virus-specific CD4^+^Foxp3^+^Treg cells. When HDAC is inhibited, SOCS1 and SOCS3 are acetylated and chromatin is relaxed, permitting virus transcription and replication and anterograde transport and shedding of HSV-1 in a lytic cycle of infection. Modulation of SOCS1–SOCS3 expression is a potential strategy for the treatment of not only viral infections but also inflammatory diseases.

## Conflict of Interest Statement

The author declares that the research was conducted in the absence of any commercial or financial relationships that could be construed as a potential conflict of interest.

## References

[1] Boehm U Klamp T Groot M Howard JC Cellular responses to interferon-gamma. Annu Rev Immunol (1997) 15:749–95 10.1146/annurev.immunol.15.1.749 9143706

[2] Fenyves AM Behrens J Spanel-Borowski K Cultured microvascular endothelial cells (MVEC) differ in cytoskeleton, expression of cadherins and fibronectin matrix. A study under the influence of interferon-gamma. J Cell Sci (1993) 106:879–90 8308070 10.1242/jcs.106.3.879

[3] Cantin EM Hinton DR Chen J Openshaw H Gamma interferon expression during acute and latent nervous system infection by herpes simplex virus type 1. J Virol (1995) 69(8):4898–905 7609058 10.1128/jvi.69.8.4898-4905.1995 PMC189304

[4] Cathcart HM Zheng M Covar JJ Liu Y Podolsky R Atherton SS Interferon-gamma, macrophages, and virus spread after HSV-1 injection. Invest Ophthalmol Vis Sci (2011) 52(7):3984–93 10.1167/iovs.10-6449 21345992 PMC3175943

[5] Valente G Ozmen L Novelli F Geuna M Palestro G Forni G Distribution of interferon-γ receptor in human tissues. Eur J Immunol (1992) 22:2403–12 10.1002/eji.1830220933 1387613

[6] Brivanlou AH Darnell JE Jr Signal transduction and the control of gene expression. Science (2002) 295(5556):813–8 10.1126/science.1066355 11823631

[7] Johnson HM Noon-Song EN Kemppainen K Ahmed CM Steroid-like signalling by interferons: making sense of specific gene activation by cytokines. Biochem J (2012) 443(2):329–38 10.1042/BJ20112187 22452815 PMC3509199

[8] Rahn E Petermann P Hsu M Rixon FJ Knebel-Morsdorf D Entry pathways of herpes simplex virus type 1 into human keratinocytes are dynamin- and cholestrol-dependent. PLoS One (2011) 6(10):e25464 10.1371/journal.pone.0025464 22022400 PMC3192061

[9] Clement C Tiwari V Scanlan PM Valyi-Nagy T Yue BYJT Shukla D A novel role for phagocytosis-like uptake in herpes simplex virus entry. J Cell Biol (2006) 174:1009–21 10.1083/jcb.200509155 17000878 PMC2064392

[10] Karasneh GA Shukla D Herpes simplex virus infects most cell types in vitro: clues to its success. Virol J (2011) 8:481 10.1186/1743-422X-8-481 22029482 PMC3223518

[11] Newcomb WW Brown JC Uncoating the herpes simplex virus genome. J Mol Biol (2007) 370(4):633–42 10.1016/j.jmb.2007.05.023 17540405 PMC1975772

[12] Lyman MG Enquist LW Herpesvirus interactions with the host cytoskeleton. J Virol (2009) 83(5):2058–66 10.1128/JVI.01718-08 18842724 PMC2643721

[13] Dodding MP Way M Coupling viruses to dynein and kinesin-1. EMBO J (2011) 30(17):3527–39 10.1038/emboj.2011.283 21878994 PMC3181490

[14] Sodeik B Ebersold MW Helenius A Microtubule-mediated transport of incoming herpes simplex virus 1 capsids to the nucleus. J Cell Biol (1997) 136(5):1007–21 10.1083/jcb.136.5.1007 9060466 PMC2132479

[15] Douglas MW Diefenbach RJ Homa FL Miranda-Saksena M Rixon FJ Vittone V Herpes simplex virus type 1 capsid protein VP26 interacts with dynein light chains RP3 and Tctex1 and plays a role in retrograde cellular transport. J Biol Chem (2004) 279(27):28522–30 10.1074/jbc.M311671200 15117959

[16] Copeland AM Newcomb WW Brown JC Herpes simplex virus replication: roles of viral proteins and nucleoporins in capsid-nucleus attachment. J Virol (2009) 83(4):1660–8 10.1128/JVI.01139-08 19073727 PMC2643781

[17] Johnson PA Everett RD The control of herpes simplex type-1 late gene transcription: a ‘TATA-box’/cap site region is sufficient for fully efficient regulated activity. Nucleic Acids Res (1986) 14(21):8247–64 10.1093/nar/14.21.8247 3024102 PMC311857

[18] Strain AK Rice SA Phenotypic suppression of a herpes simplex virus 1 ICP27 mutation by enhanced transcription of the mutant gene. J Virol (2011) 85(11):5685–90 10.1128/JVI.00315-11 21411532 PMC3094987

[19] Mears WE Rice SA The herpes simplex virus immediate-early protein ICP27 shuttles between nucleus and cytoplasm. Virology (1998) 242(1):128–37 10.1006/viro.1997.9006 9501050

[20] Huang CJ Wagner EK The herpes simplex virus type 1 major capsid protein (VP5-UL19) promoter contains two cis-acting elements influencing late expression. J Virol (1994) 68(9):5738–47 8057455 10.1128/jvi.68.9.5738-5747.1994 PMC236977

[21] Zhao L Zheng C The first identified nucleocytoplasmic shuttling herpesviral capsid protein: herpes simplex virus type 1 VP19C. PLoS One (2012) 7(8):e41825 10.1371/journal.pone.0041825 22927916 PMC3425543

[22] Lee GE Murray JW Wolkoff AW Wilson DW Reconstitution of herpes simplex virus microtubule-dependent trafficking in vitro. J Virol (2006) 80(9):4264–75 10.1128/JVI.80.9.4264 16611885 PMC1472043

[23] Wisner TW Johnson DC Redistribution of cellular and herpes simplex virus proteins from the trans-golgi network to cell junctions without enveloped capsids. J Virol (2004) 78(21):11519–35 10.1128/JVI.78.21.11519 15479793 PMC523281

[24] Antinone SE Smith GA Retrograde axon transport of herpes simplex virus and pseudorabies virus: a live-cell comparative analysis. J Virol (2010) 84(3):1504–12 10.1128/JVI.02029-09 19923187 PMC2812336

[25] Szpankowski L Encalada SE Goldstein LSB Subpixel colocalization reveals amyloid precursor protein-dependent kinesin-1 and dynein association with axonal vesicles. Proc Natl Acad Sci U S A (2012) 109(22):8582–7 10.1073/pnas.1120510109 22582169 PMC3365224

[26] Ibiricu I Maurer UE Grünewald K Characterization of herpes simplex virus type 1L-particle assembly and egress in hippocampal neurones by electron cryo-tomography. Cell Microbiol (2013) 15(2):285–91 10.1111/cmi.12093 23253400 PMC3638362

[27] Wurdinger T Gatson NN Balaj L Kaur B Breakefield XO Pegtel DM Extracellular vesicles and their convergence with viral pathways. Adv Virol (2012) 2012:767694 10.1155/2012/767694 22888349 PMC3410301

[28] Block TM Spivack JG Steiner I Deshmane S McIntosh MT Lirette RP A herpes simplex virus type 1 latency-associated transcript mutant reactivates with normal kinetics from latent infection. J Virol (1990) 64(7):3417–26 2161947 10.1128/jvi.64.7.3417-3426.1990 PMC249597

[29] McLauchlan J Rixon FJ Characterization of enveloped tegument structures (L particles) produced by alphaherpesviruses: integrity of the tegument does not depend on the presence of capsid or envelope. J Gen Virol (1992) 73:269–76 10.1099/0022-1317-73-2-269 1311356

[30] Rixon FJ Addison C McLauchlan J Assembly of enveloped tegument structures (L particles) can occur independently of virion maturation in herpes simplex virus type 1-infected cells. J Gen Virol (1992) 73:277–84 10.1099/0022-1317-73-2-277 1311357

[31] Théry C Regnault A Garin J Wolfers J Zitvogel L Ricciardi-Castagnoli P Molecular characterization of dendritic cell-derived exosomes. Selective accumulation of the heat shock protein hsc73. J Cell Biol (1999) 147(3):599–610 10.1083/jcb.147.3.599 10545503 PMC2151184

[32] Schröder NWJ Morath S Alexander C Hamann L Hartung T Zähringer U Lipoteichoic acid (LTA) of Streptococcus pneumoniae and Staphylococcus aureus activates immune cells via Toll-like receptor (TLR)-2, lipopolysaccharide-binding protein (LBP), and CD14, whereas TLR-4 and MD-2 are not involved. J Biol Chem (2003) 278(18):15587–94 10.1074/jbc.M212829200 12594207

[33] Frausto-Del-Rio D Soto-Cruz I Garay-Canales C Ambriz X Solderila G Carretero-Ortega J Interferon gamma induces actin polymerization, Rac1 activation and doen regulates phagocytosis in human monocytic cells. Cytokine (2012) 57(1):158–68 10.1016/j.cyto.2011.11.008 22137120

[34] Quetglas JI Hernáez B Galindo I Muñoz-Moreno R Cuesta-Geijo MA Alonso C Small rho GTPases and cholesterol biosynthetic pathway intermediates in African swine fever virus infection. J Virol (2012) 86:1758–67 10.1128/JVI.05666-11 22114329 PMC3264358

[35] Barois N Forquet F Davoust J Actin microfilaments control the MHC class II antigen presentation pathway in B cells. J Cell Sci (1998) 111:1791–800 9625742 10.1242/jcs.111.13.1791

[36] Vascotto F Lankar D Faure-André G Vargas P Diaz J Le Roux D The actin-based motor protein myosin II regulates MHC class II trafficking and BCR-driven antigen presentation. J Cell Biol (2007) 176(7):1007–19 10.1083/jcb.200611147 17389233 PMC2064085

[37] Wubbolts R Fernandez-Borja M Jordens I Reits E Dusseljee S Echeverri C Opposing motor activities of dynein and kinesin determine retention and transport of MHC class II-containing compartments. J Cell Sci (1999) 112:785–95 10036229 10.1242/jcs.112.6.785

[38] Hu Y Xia H Laurence B Ivashkiv LB IFN-γ and STAT1 arrest monocyte migration and modulate RAC/CDC42 pathways. J Immunol (2008) 180(12):8057–65 18523269 10.4049/jimmunol.180.12.8057 PMC2742170

[39] Heasman SJ Ridley AJ Mammalian Rho GTPases: new insights into their functions from in vivo studies. Nat Rev Mol Cell Biol (2008) 9(9):690–701 10.1038/nrm2476 18719708

[40] Utech M Ivanov AI Samarin SN Bruewer M Turner JR Mrsny RJ Mechanism of IFN-γ-induced endocytosis of tight junction proteins: myosin II-dependent vacuolarization of the apical plasma membrane. Mol Biol Cell (2005) 16:5040–52 10.1091/mbc.E05 16055505 PMC1237102

[41] Frey KG Ahmed CMI Dabelic R Jager LD Noon-Song EN Haider SM HSV-1-induced SOCS-1 expression in keratinocytes: use of a SOCS-1 antagonist to block a novel mechanism of viral immune evasion. J Immunol (2009) 183(2):1253–62 10.4049/jimmunol.0900570 19542368 PMC2706942

[42] Yokota S Yokosawa N Okabayashi T Suzutani T Fujii N Induction of supressor of cytokine signaling-3 by herpes simplex virus type 1 confers efficient viral replication. Virology (2005) 338(1):173–81 10.1016/j.virol.2005.04.028 15939448

[43] Madonna S Scarponi C Sestito R Pallotta S Cavani A Albanesi C The IFN-gamma-dependent suppressor of cytokine signaling 1 promoter activity is positively regulated by IFN regulatory factor-1 and Sp1 but repressed by growth factor independence-1b and Krüppel-like factor-4, and it is dysregulated in psoriatic keratinocytes. J Immunol (2010) 185:2467–81 10.4049/jimmunol.1001426 20644166

[44] Vuong BQ Arenzana TL Showalter BM Losman J Chen XP Mostecki J SOCS-1 localizes to the microtubule organizing complex-associated 20S proteasome. Mol Cell Biol (2004) 24(20):9092–101 10.1128/MCB.24.20.9092 15456882 PMC517868

[45] Nishi M Akihide R Tsurutani N Ohba K Sawasaki T Morishita R Requirement for microtubule integrity in the SOCS1-mediated intracellular dynamics of HIV-1 Gag. FEBS Lett (2009) 583(8):1243–50 10.1016/j.febslet.2009.03.041 19327355

[46] Stevenson NJ McFarlane C Ong ST Nahlik K Kelvin A Addley MR Suppressor of cytokine signalling (SOCS) 1 and 3 enhance cell adhesion and inhibit migration towards the chemokine eotaxin/CCL11. FEBS Lett (2010) 584:4469–74 10.1016/j.febslet.2010.10.007 20934424

[47] Zaichick SV Bohannon KP Smith GA Alphaherpesviruses and the cytoskeleton in neuronal infections. Viruses (2011) 3(7):941–81 10.3390/v3070941 21994765 PMC3185784

[48] Federici M Giustizieri ML Scarponi C Girolomoni G Alabanesi C Impaired IFN-g-dependent inflammatory responses in human keratinocytes overexpressing the suppressor of cytokine signaling 1. J Immunol (2002) 169(1):434–42 12077274 10.4049/jimmunol.169.1.434

[49] Everding B Wilhelm S Averesche S Scherdin U Holzel F Steffen M IFN-g-Induced change in microtubule organization and α-tubulin expression during growth inhibition of lung squamous carcinoma cells. J Interferon Cytokine Res (2000) 20:983–90 10.1089/10799900050198426 11096455

[50] Zou T Ouyang L Chen L Dong W Qiao H Liu Y The role of microtubule-associated protein 1S in SOCS3 regulation of IL-6 signaling. FEBS Lett (2008) 582:4015–22 10.1016/j.febslet.2008.10.055 19027008

[51] Niemand C Nimmesgern A Haan S Fischer P Scaper F Rossaint R Activation of STAT3 by IL-6 and IL-10 in primary human macrophages is differentially modulated by supressor of cytokine signaling 3. J Immunol (2003) 170(6):3263–72 12626585 10.4049/jimmunol.170.6.3263

[52] WuDunn D Spear PG Initial interaction of herpes simplex with cells is binding to heparan sulfate. J Virol (1989) 63:52–8 2535752 10.1128/jvi.63.1.52-58.1989 PMC247656

[53] Rajcani J Durmanova V Mechanisms of replication of alpha- and betaherpesviruses and their pathogenesis. Bratisl Lek Listy (2001) 102(11):505–14 11901707

[54] Dingwell KS Doering LC Johnson DC Glycoproteins E and I facilitate neuron-to-neuron spread of herpes simplex virus. J Virol (1995) 69(11):7087–98 7474128 10.1128/jvi.69.11.7087-7098.1995 PMC189628

[55] Honess RW Roizman B Regulation of herpesvirus macromolecular synthesis I. Cascade regulation of the synthesis of three groups of viral proteins. J Virol (1974) 14(1):8–19 4365321 10.1128/jvi.14.1.8-19.1974 PMC355471

[56] Nicoll MP Proença JT Efstathiou S The molecular basis of herpes simplex virus latency. FEMS Microbiol Rev (2012) 36:684–705 10.1111/j.1574-6976.2011.00320.x 22150699 PMC3492847

[57] Wysocka J Herr W The herpes simplex virus VP16-induced complex: the makings of a regulatory switch. Trends Biochem Sci (2003) 28(6):294–304 10.1016/S0968-0004(03)00088-4 12826401

[58] Umbach JL Kramer MF Jurak I Karnowski HW Coen DM Cullen BR MicroRNAs expressed by herpes simplex virus 1 during latent infection regulate viral mRNAs. Nature (2008) 454(7205):780–3 10.1038/nature07103 18596690 PMC2666538

[59] Wagner EK Bloom DC Experimental investigation of herpes simplex virus latency. Clin Microbiol Rev (1997) 10(3):419–43 9227860 10.1128/cmr.10.3.419 PMC172928

[60] Zabolotny JM Krummenacher C Fraser NW Zabolotny JM Krummenacher C Fraser NW Latency-associated transcript is a stable intron which branches at a guanosine. J Virol (1997) 71(6):4199–208 9151806 10.1128/jvi.71.6.4199-4208.1997 PMC191634

[61] Doerig C Pizer LI Wilcox CL An antigen encoded by the latency-associated transcript in neuronal cell cultures latently infected with herpes simplex virus type 1. J Virol (1991) 65(5):2724–7 1850045 10.1128/jvi.65.5.2724-2727.1991 PMC240637

[62] Javier RT Stevens JG Dissette VB Wagner EK A herpes simplex virus transcript abundant in latently infected neuron is dispensable for establishment of the latent state. Virology (1988) 166:254–57 2842950 10.1016/0042-6822(88)90169-9

[63] Thompson RL Sawtell NM The herpes simplex virus type 1 latency-associated transcript gene regulates the establishment of latency. J Virol (1997) 71(7):5432–40 9188615 10.1128/jvi.71.7.5432-5440.1997 PMC191783

[64] Nicoll MP Efstathiou S Expression of the herpes simplex virus type 1 latency-associated transcripts does not influence latency establishment of virus mutants deficient for neuronal replication. J Gen Virol (2013) 94:2489–94 10.1099/vir.0.056176-0 23907392 PMC3809108

[65] Marshall KR Lachmann RH Efstathiou S Rinaldi A Preston CM Long-term transgene expression in mice infected with a herpes simplex virus type 1 mutant severely impaired for immediate-early gene expression. J Virol (2000) 74(2):956–64 10.1128/JVI.74.2.956-964.2000 10623758 PMC111616

[66] Miller CS Danaher RJ Jacob RJ Molecular aspects of herpes simplex virus I latency, reactivation, and recurrence. Crit Rev Oral Biol Med (1998) 9(4):541–62 10.1177/10454411980090040901 9825226

[67] Lillycrop KA Dawson SJ Estridge JK Gerster T Matihias P Latchmanl DS Repression of a herpes simplex virus immediate-early promoter by the Oct-2 transcription factor is dependent on an inhibitory region at the N terminus of the protein. Mol Cell Biol (1994) 14(11):7633–42 7935477 10.1128/mcb.14.11.7633-7642.1994 PMC359299

[68] Hagmann M Georgiev O Schaffner W The VP16 paradox: herpes simplex virus VP16 contains a long-range activation domain but within the natural multiprotein complex activates only from promoter-proximal positions. J Virol (1997) 71(8):5952–62 9223485 10.1128/jvi.71.8.5952-5962.1997 PMC191851

[69] Kolb G Kristie TM Association of the cellular coactivator HCF-1 with the Golgi apparatus in sensory neurons. J Virol (2008) 82:9555–63 10.1128/JVI.01174-08 18667495 PMC2546983

[70] Sawtell NM Thompson RL Rapid in vivo reactivation of herpes simplex virus in latently infected murine ganglionic neurons after transient hyperthermia. J Virol (1992) 66(4):2150–6 1312625 10.1128/jvi.66.4.2150-2156.1992 PMC289007

[71] Umbach JL Nagel MA Cohrs RJ Gilden DH Cullen BR Analysis of human alphaherpesvirus microRNA expression in latently infected human trigeminal ganglia. J Virol (2009) 83(20):10677–83 10.1128/JVI.01185-09 19656888 PMC2753103

[72] Bishop GA Marlin SD Schwartz SA Glorioso JC Human natural killer cell recognition of herpes simplex virus type 1 glycoproteins: specificity analysis with the use of monoclonal antibodies and antigenic variants. J Immunol (1984) 133(4):2206–14 6206157

[73] Malmgaard L Melchjorsen J Bowie AG Mogensen SC Paludan SR Viral activation of macrophages through TLR-dependent and -independent pathways. J Immunol (2004) 173:6890–8 15557184 10.4049/jimmunol.173.11.6890

[74] Sato A Linehan MM Iwasaki A Dual recognition of herpes simplex viruses by TLR2 and TLR9 in dendritic cells. Proc Natl Acad Sci U S A (2006) 103(46):17343–8 10.1073/pnas.0605102103 17085599 PMC1859932

[75] Green JA Yeh T Overall JC Jr Sequential production of IFN-alpha and immune specific IFN-gamma by human mononuclear leukocytes exposed to herpes simplex virus. J Immunol (1981) 127(3):1192–6 6167619

[76] Wang JP Bowen GN Zhou S Cerny A Zacharia A Knipe DM Role of specific innate immune responses in herpes simplex virus infection of the central nervous system. J Virol (2012) 86(4):2273–81 10.1128/JVI.06010-11 22171256 PMC3302371

[77] Sørensen LN Reinert LS Malmgaard L Bartholdy C Thomsen AR Søren R TLR2 and TLR9 synergistically control herpes simplex virus infection in the brain. J Immunol (2008) 181(12):8604–12 19050280 10.4049/jimmunol.181.12.8604

[78] Loret S Guay G Lippé R Comprehensive characterization of extracellular herpes simplex virus type 1 virions. J Virol (2008) 82(17):8605–18 10.1128/JVI.00904-08 18596102 PMC2519676

[79] Knickelbein JE Khanna KM Yee MB Baty CJ Kinchington PR Hendricks RL Noncytotoxic lytic granule-mediated CD8^+^ T cell inhibition of HSV-1 reactivation from neuronal latency. Science (2008) 322:268–71 10.1126/science.1164164 18845757 PMC2680315

[80] Theil D Derfuss T Paripovic I Herberger S Meinl E Schueler O Short communication: ganglia causes chronic immune response. Am J Pathol (2003) 163(6):2179–84 10.1016/S0002-9440(10)63575-4 14633592 PMC1892378

[81] Feldman LT Ellison AR Voytek CC Yang L Krause P Margolis TP Spontaneous molecular reactivation of herpes simplex virus type 1 latency in mice. Proc Natl Acad Sci U S A (2002) 99(2):978–83 10.1073/pnas.022301899 11773630 PMC117416

[82] Liu T Tang Q Hendricks RL Inflammatory infiltration of the trigeminal ganglion after herpes simplex virus type 1 corneal infection. J Virol (1996) 70(1):264–71 8523535 10.1128/jvi.70.1.264-271.1996 PMC189813

[83] Suvas S Azkur AK Rouse BT Qa-1b and CD94-NKG2a interaction Regulate cytolytic activity of herpes simplex virus-specific memory CD8? T cells in the latently infected trigeminal ganglia. J Immunol (2006) 176:1703–11 16424200 10.4049/jimmunol.176.3.1703

[84] Long EO Negative signalling by inhibitory receptors: the NK cell paradigm. Immunol Rev (2008) 224:70–84 10.1111/j.1600-065X.2008.00660.x 18759921 PMC2587243

[85] Turnley AM Starr R Bartlett PF Failure of sensory neurons to express class I MHC is due to differential SOCS1 expression. J Neuroimmunol (2002) 123:35–40 10.1016/S0165-5728(01)00480-5 11880147

[86] Strahl BD Allis CD The language of covalent histone modifications. Nature (2000) 403:41–5 10.1038/47412 10638745

[87] Guise AJ Budayeva HG Diner BA Cristea IM Histone deacetylases in herpesvirus replication and virus-stimulated host defense. Viruses (2013) 5:1607–32 10.3390/v5071607 23807710 PMC3738950

[88] Xiong H Du W Zhang YJ Hong J Su WY Tang JT Trichostatin A, a histone deacetylase inhibitor, suppresses JAK2/STAT3 signaling via inducing the promoter-associated histone acetylation of SOCS1 and SOCS3 in human colorectal cancer cells. Mol Carcinog (2012) 51:174–84 10.1002/mc.20777 21520296

[89] Bertke AS Swanson SM Chen J Imai Y Kinchington PR Margolis TP A5-positive primary sensory neurons are nonpermissive for productive infection with herpes simplex virus 1 in vitro. J Virol (2011) 85(13):6669–77 10.1128/JVI.00204-11 21507969 PMC3126511

[90] Gao B Wang Y Xu W Li S Li Q Xiong S Inhibition of histone deacetylase activity suppresses IFN-γ induction of tripartite motif 22 via CHIP-mediated proteasomal degradation of IRF-1. J Immunol (2013) 191:464–71 10.4049/jimmunol.1203533 23729439

[91] Qin H Holdbrooks AT Liu Y Reynolds SL Yanagisawa LL Benveniste EN SOCS3 deficiency promotes M1 macrophage polarization and inflammation. J Immunol (2012) 189(7):3439–48 10.4049/jimmunol.1201168 22925925 PMC4184888

[92] Qin H Yeh WI De Sarno P Holdbrooks AT Liu Y Muldowney MT Signal transducer and activator of transcription-3/suppressor of cytokine signaling-3 (STAT3/SOCS3) axis in myeloid cells regulates neuro inflammation. Proc Natl Acad Sci U S A (2012) 109:5004–9 10.1073/pnas.1117218109 22411837 PMC3323949

